# Supplementary material for: Interplay between Pleiotropy and Secondary Selection Determines Rise and Fall of Mutators in Stress Response
Source: PLoS Comput Biol. 2010 Mar 12;6(3):e1000710. doi: 10.1371/journal.pcbi.1000710 (PMC2837395; doi:10.1371/journal.pcbi.1000710)
Supplement: Figure S1 — Genotypic mutations: the main mechanism of adaptation. In order to elucidate the main cause of increase in fitness upon adaptation, we investigated the time progression of microscopic physical-chemical quantities of RCGs, which determine fitness according to Eq.(1). We plot the concentration of complexes between protein 2 and 3 (F 23, black curve), the total concentrations of the protein 2 (C 2, red curve) and 3 (C 3, blue curve) in the top panels, the fractional concentration of protein 1 in its functional (monomeric) form (F 1/C 1) in the second panels, thermal probabilities to form functional complex between protein 2 and 3 (P23int) in the third panels, and stabilities of proteins 1 (black), 2 (red) and 3 (blue) (P not) in the bottom panels. The green lines at t = 20000 mark the time of stresses, which are (A) temperature increase, (B) 3 fold decrease of the base birth rate (b 0) (stationary phase), and (C) 10 fold drop of the optimal total concentration (C 0) of all proteins (starvation). The fractional concentrations of proteins are determined by the total concentrations of proteins and the binding constants between all possible pairs of proteins in a cell according to the LMA equations as explained in the Main text. Changes in all of these quantities are due to genotypic mutations, which change the properties of proteins such as their stability, solubility (affecting F 1 and F 23) and propensity for functional interaction P23int, independent on the phenotypic changes of the total protein concentrations. (0.26 MB DOC) [file pcbi.1000710.s001.doc]

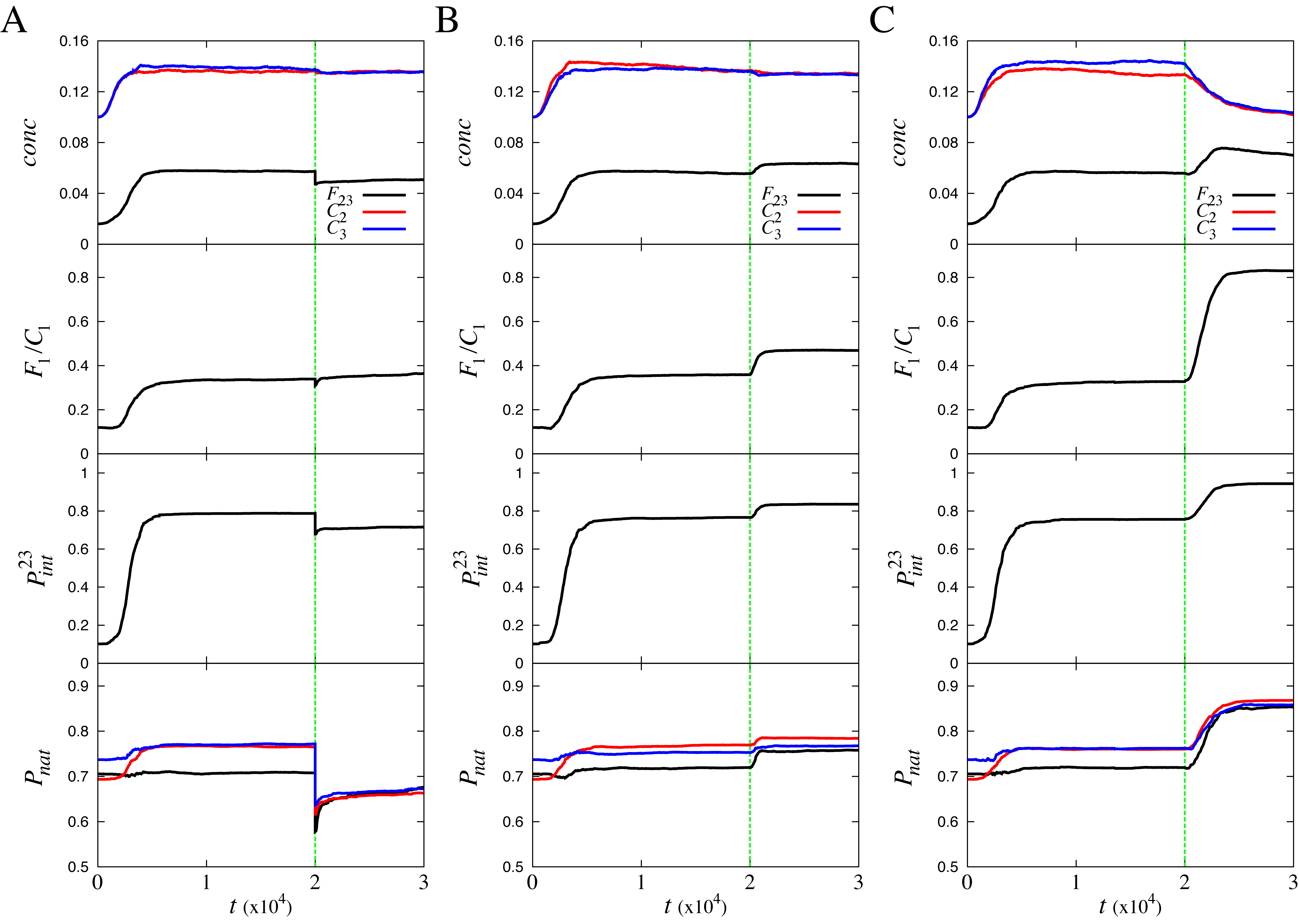


**Figure S1. Genotypic mutations: the main mechanism of adaptation.** In order to elucidate the main cause of increase in fitness upon adaptation, we investigated the time progression of microscopic physical-chemical quantities of RCGs, which determine fitness according to Eq.(1). We plot the concentration of complexes between protein 2 and 3 (*F*_23_, black curve), the total concentrations of the protein 2 (*C*_2_, red curve) and 3 (*C*_3_, blue curve) in the top panels, the fractional concentration of protein 1 in its functional (monomeric) form () in the second panels, thermal probabilities to form functional complex between protein 2 and 3 () in the third panels, and stabilities of proteins 1 (black), 2 (red) and 3 (blue) () in the bottom panels. The green lines at *t*=20000 mark the time of stresses, which are (A) temperature increase, (B) 3 fold decrease of the base birth rate (*b*_0_) (stationary phase), and (C) 10 fold drop of the optimal total concentration (*C*_0_) of all proteins (starvation). The fractional concentrations of proteins are determined by the total concentrations of proteins and the binding constants between all possible pairs of proteins in a cell according to the LMA equations as explained in the Main text. Changes in all of these quantities are due to genotypic mutations, which change the properties of proteins such as their stability, solubility (affecting ) and propensity for functional interaction , independent on the phenotypic changes of the total protein concentrations.
